# Excesso de Pressão Arterial Sistólica Associada com Poluição Aérea por Material Particulado Fino Acima da Diretriz da OMS no Brasil

**DOI:** 10.36660/abc.20230347

**Published:** 2023-12-06

**Authors:** Pedro Rafael Vieira de Oliveira Salerno, Issam Motairek, Luis Augusto Palma Dallan, Brendan Bourges-Sevenier, Sanjay Rajagopalan, Sadeer G. Al-Kindi

**Affiliations:** 1 Harrington Heart and Vascular Institute University Hospitals Cleveland Medical Center Cleveland Ohio EUA Harrington Heart and Vascular Institute , University Hospitals Cleveland Medical Center , Cleveland , Ohio – EUA; 2 Case Western Reserve University School of Medicine Cleveland Ohio EUA Case Western Reserve University School of Medicine , Cleveland , Ohio – EUA

**Keywords:** Poluição do Ar, Pressão Arterial, Meio Ambiente, Saúde Pública

## Introdução

A doença cardiovascular continua sendo a principal causa mundial de mortalidade, e a hipertensão tem sido implicada como tendo a maior associação de causação entre todos os fatores de risco de doenças cardiovasculares. ^1^ Assim, não é de surpreender que a hipertensão seja o principal fator de risco de morte e a segunda maior causa de anos de vida ajustados por incapacidade no Brasil, fortemente impactando sua população e o sistema nacional de saúde. ^2^ A hipertensão também está diretamente ligada à poluição do ar, especialmente ao material particulado fino < 2,5 mícrons (PM _2,5_ ), que tem sido identificado como um fator de risco independente para aumento da pressão arterial sistólica (PAS). ^3^ No Brasil, como na maioria dos países, a principal fonte de exposição à poluição do ar em áreas urbanas resulta principalmente da queima de combustíveis fósseis pelo trânsito de veículos e pela geração de energia industrial, enquanto, em áreas rurais do Brasil, a poluição do ar resulta principalmente da queima de biomassa. ^4^

No Brasil, foram realizados estudos anteriores investigando os efeitos da exposição ao PM _2,5_ na pressão arterial em trabalhadores ao ar livre que são mais suscetíveis à poluição do ar ambiente por emissões veiculares, como controladores de tráfego, demonstrando a ligação entre a concentração de PM _2,5_ e o aumento da PAS. ^5 , 6^ No entanto, esses estudos tendem a concentrar-se nas regiões metropolitanas e urbanas. ^5 , 6^ Assim, devido ao grande tamanho geográfico e à diversidade regional do Brasil, existe uma lacuna substancial no conhecimento em relação às áreas menos povoadas. No presente estudo, visamos estimar o excesso de PAS atribuível às concentrações de PM _2,5_ acima do limite da diretriz da Organização Mundial da Saúde (OMS) no Brasil, bem como em todos os seus estados e regiões.

## Métodos

Obtivemos as concentrações de PM _2,5_ ponderadas pela população de 2019 para os 26 estados brasileiros e o Distrito Federal a partir do estudo *Global Burden of Disease* (GBD), um esforço de colaboração internacional para quantificar os níveis e tendências na saúde e fornecer dados publicamente disponíveis. ^7^ Modelos de sistemas de monitoramento ao nível do solo e de satélite, bem como modelos de transporte químico, foram utilizados pelo GBD para estimar as concentrações de PM _2,5_ em μg/m ^3^ Os estados também foram estratificados de acordo com as regiões (Norte, Nordeste, Centro-Oeste, Sudeste e Sul) determinados pelo Instituto Brasileiro de Geografia e Estatística (IBGE) ^9^ e de acordo com quartis do Índice Sociodemográfico (SDI). ^7^ O SDI, uma ferramenta desenvolvida pelo GBD, é um indicador composto de condições socioeconômicas que afetam os desfechos de saúde que varia de 0 a 1. ^7^ Uma faixa de exposição ao PM _2,5_ e da resposta da PAS foi obtida a partir de uma metanálise de estudos observacionais (aumento de 0,06 mmHg na PAS por aumento de 1 µg/m ^3^ na concentração de PM _2,5_ ) e de uma revisão de ensaios de filtração de ar (0,19 mmHg por aumento de 1 µg/m ^3^ na concentração de PM _2,5_ ), ^10 , 11^ resultando assim em uma faixa de resposta à exposição de 0,06 a 0,19 mmHg por aumento de 1 μg/m ^3^ na concentração de PM _2,5_ . A metanálise incluiu 28 estudos observacionais (10 estudos de coorte e 18 estudos transversais) que examinaram a exposição de longo prazo (> 1 ano) ao PM _2,5_ e hipertensão em populações saudáveis. ^10^ A revisão sistemática incluiu um total de 10 ensaios clínicos randomizados em humanos que avaliaram filtros de ar pessoais (incluindo filtros de ar particulado de alta eficiência e precipitadores eletrostáticos) em um ambiente doméstico ou residencial, observando os efeitos da filtração versus nenhuma filtração na pressão arterial. ^11^ O excesso de PAS foi considerado como o aumento da PAS devido ao nível de concentração de PM _2,5_ acima de 5 μg/m ^3^ , com base nas atuais Diretrizes de Qualidade do Ar da OMS. ^8^ A aprovação do conselho de revisão institucional não foi necessária devido à natureza dos dados publicamente disponíveis. As análises estatísticas e os mapas foram produzidos utilizando o software de acesso aberto R v 4.1.2.

**Excesso de PAS, estimativas observacionais**


$$=\left[\frac{\left(\text{ Concentração média de }{PM}_{2,5}\text{ no país }-5\mu g/m^{3}\right)\times 0,06mmHg}{g/m^{3}}\right]$$

**Excesso de PAS, estimativas de intervenção**


$$=\left[\frac{\left(\text{ Concentração média de }{PM}_{2,5}\text{ no país }-5\mu g/m^{3}\right)\times 0,19mmHg}{g/m^{3}}\right]$$

## Resultados

**Figura 1:**
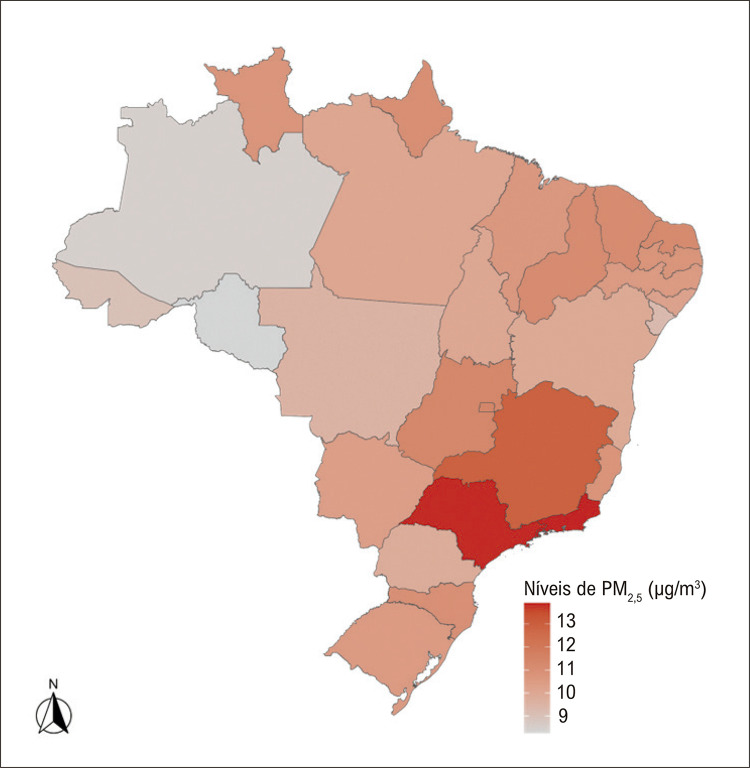
– Variação da exposição ao material particulado fino < 2,5 mícrons (PM 2,5 ) em nível estadual no Brasil. Medido em μg/m 3 .

**Figura 2:**
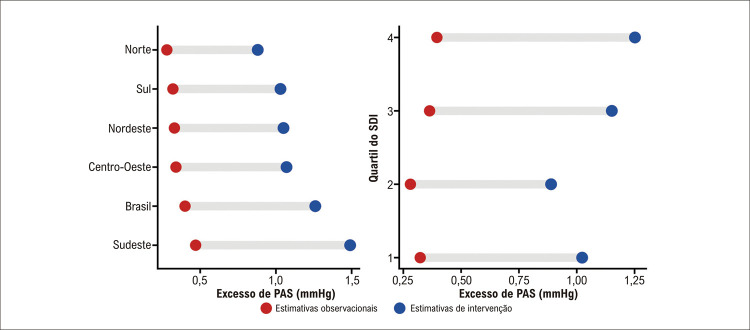
– Excesso de pressão arterial sistólica (PAS) com base em estimativas observacionais e de intervenção atribuídas à exposição ao PM 2,5 no Brasil de acordo com região e quartis do Índice Sociodemográfico (SDI).

A concentração média de PM _2,5_ ponderada pela população do Brasil foi de 10,68 μg/m ^3^ , o que resultou em uma faixa de excesso de PAS de 0,40 a 1,26 mmHg com base em estimativas observacionais e de intervenção, respectivamente. A Tabela 1 descreve a concentração média de PM _2,5_ específica por estado e o excesso de PAS em 2019. A Figura 1 mapeia a variabilidade interestadual da concentração média de PM _2,5_ , e a Figura 2 ilustra o excesso de PAS de acordo com a região e os quartis do SDI. Rio de Janeiro (0,53 a 1,69 mmHg) e São Paulo (0,53 a 1,68 mmHg) tiveram os maiores níveis de excesso de PAS, enquanto Rondônia (0,20 a 0,62 mmHg) e Amazonas (0,21 a 0,66 mmHg) tiveram os menores. Comparando os estados com carga mais baixa (Rondônia) e com carga mais alta (Rio de Janeiro), foi observada uma diferença percentual relativa de aproximadamente 90% a 92% (faixa da estimativa observacional para a de intervenção) para o excesso de PAS. As diferenças regionais e os quartis do SDI no excesso de PAS são exibidas na Figura 2 . O Sudeste (0,47 a 1,49 mmHg) foi a região que teve a maior carga de excesso de PAS, enquanto o Norte (0,28 a 0,88 mmHg) foi a região menos afetada. Em relação aos quartis do SDI, o terceiro (0,36 a 1,15 mmHg) e o quarto (0,39 a 1,25 mmHg) apresentaram a maior carga, e o segundo quartil (0,28 a 0,88 mmHg) a menor.

**Tabela 1:** – Concentração média de PM **2,5** e Índice Sociodemográfico (SDI) para os estados brasileiros em 2019 e excesso de pressão arterial sistólica (PAS) com base em estimativas observacionais e de intervenção atribuídas à exposição ao PM **2,5**

Região	Estado	SDI	Quartil do SDI	PM _**2,5**_ média (μg/m ^**3**^ )	Excesso de PAS (mmHg) Estimativas observacionais	Excesso de PAS (mmHg) Estimativas de intervenção
Norte	Acre	0,56	1	9,01	0,24	0,76
Nordeste	Alagoas	0,52	1	10,20	0,31	0,99
Norte	Amapá	0,64	3	10,97	0,36	1,13
Norte	Amazonas	0,6	2	8,47	0,21	0,66
Nordeste	Bahia	0,56	1	9,92	0,30	0,94
Nordeste	Ceará	0,56	1	11,11	0,37	1,16
Centro-Oeste	Distrito Federal	0,78	4	11,34	0,38	1,20
Sudeste	Espírito Santo	0,66	4	10,77	0,35	1,10
Centro-Oeste	Goiás	0,63	3	11,29	0,38	1,20
Nordeste	Maranhão	0,44	1	10,61	0,34	1,07
Centro-Oeste	Mato Grosso	0,64	3	9,52	0,27	0,86
Centro-Oeste	Mato Grosso do Sul	0,64	3	10,36	0,32	1,02
Sudeste	Minas Gerais	0,64	3	12,89	0,47	1,50
Norte	Pará	0,57	2	10,08	0,30	0,96
Nordeste	Paraíba	0,55	1	10,74	0,34	1,09
Sul	Paraná	0,66	4	9,79	0,29	0,91
Nordeste	Pernambuco	0,57	2	10,50	0,33	1,05
Nordeste	Piauí	0,51	1	11,11	0,37	1,16
Sudeste	Rio de Janeiro	0,7	4	13,91	0,53	1,69
Nordeste	Rio Grande do Norte	0,58	2	11,15	0,37	1,17
Sul	Rio Grande do Sul	0,68	4	10,50	0,33	1,04
Norte	Rondônia	0,61	2	8,26	0,20	0,62
Norte	Roraima	0,61	3	10,74	0,34	1,09
Sul	Santa Catarina	0,69	4	10,93	0,36	1,13
Sudeste	São Paulo	0,7	4	13,87	0,53	1,68
Nordeste	Sergipe	0,58	2	9,28	0,26	0,81
Norte	Tocantins	0,58	2	10,01	0,30	0,95
**Brasil**		0,64	3	11,65	0,40	1,26

## Discussão

No contexto da crise de hipertensão no Brasil, com 1 a cada 4 brasileiros relatando hipertensão, nossos resultados destacam a contribuição menos reconhecida da exposição ao PM _2,5_ para o aumento da PAS. ^2^ Embora a elevação da PAS no Brasil seja inferior à média global (0,40 versus 2,4 mmHg, estimativas observacionais), foram observadas disparidades interestaduais, regionais e sociodemográficas substanciais em relação à magnitude do excesso de PAS, particularmente na Região Sudeste, onde os níveis de PM _2,5_ também são os mais altos. ^8^ Além disso, o achado de que todos os estados brasileiros possuem concentrações de PM _2,5_ acima do limite atual da OMS enfatiza a necessidade de programas nacionais de poluição do ar mais fortes e coesos, especialmente nos estados do Rio de Janeiro e de São Paulo, que contêm as maiores áreas urbanas do Brasil. ^12^ Dado o impacto dos combustíveis fósseis na poluição do ar em áreas urbanas, essas iniciativas devem se concentrar na priorização do uso de fontes de energia não fósseis, bem como na melhoria da infraestrutura para apoiar sistemas maiores de transporte público e promover o uso de veículos de energia limpa. ^13^ Além disso, devido às altas concentrações de PM _2,5_ no estado de São Paulo, o maior produtor de açúcar do Brasil, deve-se também considerar o impacto da queima de cana-de-açúcar antes da colheita e da imposição de maiores restrições industriais, especialmente porque o poluição do ar durante a temporada das queimadas tem sido associada ao aumento de internações hospitalares. ^4 , 14^

Embora, no presente estudo, a Região Norte tenha a menor concentração de PM _2,5_ , outros estudos que utilizaram diferentes métodos de estimativa de PM _2,5_ descreveram uma maior carga de poluição do ar na região. ^15^ De fato, a Região Norte desempenha um papel crucial na poluição do ar no Brasil. ^16^ A queima de biomassa que ocorre na Amazônia, por exemplo, emite grandes quantidades de PM _2,5_ , que não somente impactam áreas próximas aos incêndios, mas também causam doenças e mortes prematuras em áreas distantes deles. ^16^ Além disso, a região também é descrita como um *hotspot* socioclimático, combinando altos níveis de vulnerabilidade social e ambiental, aumentando a necessidade de intervenções socioambientais e de saúde direcionadas na região. ^16^

Por último, embora o valor numérico do aumento da PAS possa parecer dispensável, deve ser visto à luz do seu impacto em um grande número de indivíduos e considerado como um componente de um continuum de exposições que têm um efeito aditivo na pressão arterial. Além disso, têm sido propostas múltiplas vias biológicas que medeiam o impacto do PM _2,5_ na pressão arterial, incluindo inflamação sistêmica, respostas oxidativas e disfunção endotelial. ^17^ Estudos recentes da China também implicam a ativação do eixo hipotálamo-hipófise-adrenal em resposta à exposição a altos níveis de PM _2,5_ , culminando em aumentos significativos nos hormônios séricos do estresse (cortisol, cortisona, epinefrina e norepinefrina). ^17 , 18^

As limitações do presente estudo incluem aquelas inerentes ao seu caráter ecológico, sua dependência de dados secundários e análise em nível estadual, que pode subestimar o efeito nas populações de áreas metropolitanas industrializadas.
