# Supplementary material for: Excesso de Pressão Arterial Sistólica Associada com Poluição Aérea por Material Particulado Fino Acima da Diretriz da OMS no Brasil
Source: Arq Bras Cardiol. 2023 Dec 6;120(11):e20230347. [Article in Portuguese] doi: 10.36660/abc.20230347 (PMC10763691; doi:10.36660/abc.20230347)
Supplement: Translation

# Translation: Excess Systolic Blood Pressure Associated with Fine Particulate Matter Air Pollution above the WHO Guidelines in Brazil

**Authors:** Pedro Rafael Vieira de Oliveira Salerno, Issam Motairek, Luis Augusto Palma Dallan, Brendan Bourges-Sevenier, Sanjay Rajagopalan, Sadeer G. Al-Kindi

**Affiliations:** 1 USA Harrington Heart and Vascular Institute, University Hospitals Cleveland Medical Center, Cleveland, Ohio – USA; 2 USA Case Western Reserve University School of Medicine, Cleveland, Ohio – USA

**Keywords:** Air Pollution, Arterial Pressure, Environment, Public Health

## Introduction

Cardiovascular disease remains the leading cause of mortality worldwide, and hypertension has been implicated as having the highest association of causation among all of the cardiovascular disease risk factors. ^1^ Thus, unsurprisingly, hypertension is the main risk factor for death and the second major cause of disability-adjusted life years in Brazil, heavily impacting its population and national healthcare system. ^2^ It is also directly linked to air pollution, especially fine particulate matter < 2.5 microns (PM _2.5_ ), which has been identified as an independent risk factor for increased systolic blood pressure (SBP). ^3^ In Brazil, as in most countries, the main source of exposure to air pollution in urban areas primarily results from the burning of fossil fuels via vehicular traffic and industrial power generation, while, in rural Brazil, air pollution mainly results from the burning of biomass as well as deforestation. ^4^

In Brazil, previous studies have been conducted investigating the effects of PM _2.5_ exposure on blood pressure in outdoor workers who are most susceptible to ambient air pollution from vehicular emissions, such as traffic controllers, demonstrating the link between PM _2.5_ concentration and increased SBP. ^5 , 6^ However, these studies tend to focus on metropolitan and urban regions. ^5 , 6^ Thus, due to Brazil’s large geographical size and regional diversity, there exists a substantial gap in knowledge in the less populated areas. In this study, we aimed to estimate the excess SBP attributable to PM _2.5_ concentrations above the threshold of the World Health Organization (WHO) guidelines in Brazil, as well as in all its states and regions.

## Methods

We obtained the 2019 population-weighted PM _2.5_ concentrations for the 26 Brazilian states and the Federal District from the Global Burden of Disease (GBD), an international collaboration effort to quantify levels and trends in health and provide publicly available data. ^7^ Both satellite and ground-level monitoring system models, as well as chemical transport models, were used by the GBD to estimate PM _2.5_ concentrations in μg/m ^3^ . ^8^ The states were also stratified according to the regions (North, Northeast, Midwest, Southeast, and South) determined by the Brazilian Institute of Geography and Statistics (IBGE) ^9^ and according to quartiles of the Socio-demographic Index (SDI). ^7^ The SDI, a tool developed by the GBD, is a composite indicator of background social and economic conditions affecting health outcomes that ranges from 0 to 1. ^7^ A range of exposure to PM _2.5_ and blood pressure response was obtained from a metanalysis of observational studies (0.06 mmHg increase in SBP per 1 µg/m ^3^ increase in PM _2.5_ concentration) and from a review of trials of air filtration (0.19 mmHg per 1 μg/m ^3^ increase in PM _2.5_ concentration), ^10 , 11^ thus resulting in an exposure response range of 0.06 to 0.19 mmHg per 1 μg/m ^3^ increase in PM _2.5_ concentration. The metanalysis included 28 observational studies (10 cohort and 18 cross-sectional studies) examining long-term exposure (> 1 year) to PM _2.5_ and hypertension in healthy populations. ^10^ The systematic review included a total of 10 randomized controlled trials in humans that evaluated personal air cleaners (including high-efficiency particulate air filters and electrostatic precipitators) in a home or residential setting observing the effects of filtration versus no filtration on blood pressure. ^11^ Excess SBP was considered as the increase in SBP due to the level of PM _2.5_ concentration above 5 μg/m ^3^ , based on the current WHO Air Quality Guidelines. ^8^ Institutional review board approval was not required due to the nature of the publicly available data. Statistical analyses and maps were produced using the open-access software R v 4.1.2.

**Excess SBP, observational estimates**


$$=\left[\frac{\left(\text{ Mean Country }{PM}_{2,5}\text{ Concentration }-5\mu g/m^{3}\right)\times 0,06mmHg}{g/m^{3}}\right]$$

**Excess SBP, interventional estimates**


$$=\left[\frac{\left(\text{ Mean Country }{PM}_{2,5}\text{ Concentration }-5\mu g/m^{3}\right)\times 0,19mmHg}{g/m^{3}}\right]$$

## Results

The mean population-weighted PM _2.5_ concentration for Brazil was 10.68 μg/m ^3^ , which resulted in an excess SBP range of 0.40 to 1.26 mmHg based on observational and interventional estimates, respectively. Table 1 describes the state-specific mean PM _2.5_ concentration and excess SBP in 2019. Figure 1 maps the inter-state variability of the mean PM _2.5_ concentration, and Figure 2 illustrates the excess SBP according to region and SDI quartiles. Rio de Janeiro (0.53 to 1.69 mmHg) and São Paulo (0.53 to 1.68 mmHg) had the highest excess SBP, while Rondônia (0.20 to 0.62 mmHg) and Amazonas (0.21 to 0.66 mmHg) had the lowest. Comparing the lowest (Rondônia) to the highest (Rio de Janeiro) burden states, a relative percent difference of approximately 90% to 92% (observational to interventional range) was observed for excess SBP. Regional and SDI quartile differences in excess SBP are shown in Figure 2 . The Southeast (0.47 to 1.49 mmHg) had the highest excess SBP burden, while the North (0.28 to 0.88 mmHg) was the least affected. Concerning SDI quartiles, the third (0.36 to 1.15 mmHg) and fourth (0.39 to 1.25 mmHg) quartiles observed the highest burden, and the second quartile (0.28 to 0.88 mmHg) the lowest.

**Table 1:** – Mean PM **2.5** concentration and Socio-demographic Index (SDI) for Brazilian states in 2019 and excess systolic blood pressure (SBP) based on observational and interventional estimates attributed to PM **2.5** exposure

Region	State	SDI	SDI quartile	Mean PM _**2.5**_ (μg/m ^**3**^ )	Excess SBP (mmHg) Observational estimates	Excess SBP (mmHg) Interventional Estimates
North	Acre	0.56	1	9.01	0.24	0.76
Northeast	Alagoas	0.52	1	10.20	0.31	0.99
North	Amapá	0.64	3	10.97	0.36	1.13
North	Amazonas	0.6	2	8.47	0.21	0.66
Northeast	Bahia	0.56	1	9.92	0.30	0.94
Northeast	Ceará	0.56	1	11.11	0.37	1.16
Midwest	Distrito Federal	0.78	4	11.34	0.38	1.20
Southeast	Espírito Santo	0.66	4	10.77	0.35	1.10
Midwest	Goiás	0.63	3	11.29	0.38	1.20
Northeast	Maranhão	0.44	1	10.61	0.34	1.07
Midwest	Mato Grosso	0.64	3	9.52	0.27	0.86
Midwest	Mato Grosso do Sul	0.64	3	10.36	0.32	1.02
Southeast	Minas Gerais	0.64	3	12.89	0.47	1.50
North	Pará	0.57	2	10.08	0.30	0.96
Northeast	Paraíba	0.55	1	10.74	0.34	1.09
South	Paraná	0.66	4	9.79	0.29	0.91
Northeast	Pernambuco	0.57	2	10.50	0.33	1.05
Northeast	Piauí	0.51	1	11.11	0.37	1.16
Southeast	Rio de Janeiro	0.7	4	13.91	0.53	1.69
Northeast	Rio Grande do Norte	0.58	2	11.15	0.37	1.17
South	Rio Grande do Sul	0.68	4	10.50	0.33	1.04
North	Rondônia	0.61	2	8.26	0.20	0.62
North	Roraima	0.61	3	10.74	0.34	1.09
South	Santa Catarina	0.69	4	10.93	0.36	1.13
Southeast	São Paulo	0.7	4	13.87	0.53	1.68
Northeast	Sergipe	0.58	2	9.28	0.26	0.81
North	Tocantins	0.58	2	10.01	0.30	0.95
**Brazil**		0.64	3	11.65	0.40	1.26

**Figure 1:** – Particulate matter 2.5 (PM 2.5 ) state-level exposure variation in Brazil. Measured in μg/m 3 .

**Figure 2:** – Excess systolic blood pressure (SBP) based on observational and interventional estimates attributed to PM 2.5 exposure in Brazil according to region and Socio-demographic Index (SDI) quartiles.

## Discussion

In the context of the Brazilian hypertension crisis, with 1 in every 4 Brazilians reporting hypertension, our findings highlight the less recognized contribution of PM _2.5_ exposure to increased SBP. ^2^ Although Brazil’s SBP elevation is lower than the global average (0.40 versus 2.4 mmHg, observational estimates), substantial inter-state, regional, and socio-demographic disparities regarding the magnitude of excess SBP were observed, particularly in the Southeast Region, where PM _2.5_ levels are also the highest. ^8^ In addition, the finding that all Brazilian states possess PM _2.5_ concentrations above the current WHO threshold emphasizes the need for stronger cohesive nationwide air pollution programs, especially in the southeastern states of Rio de Janeiro and São Paulo, which contain the largest urban areas in Brazil. ^12^ Given the impact of fossil fuels on air pollution in urban areas, these initiatives should focus on prioritizing the use of non-fossil energy sources, as well as improving infrastructure to support larger public transport systems and promote the use of clean energy vehicles. ^13^ Additionally, due to high PM _2.5_ concentrations in São Paulo, Brazil’s largest sugar-producing state, one must also consider the impact of pre-harvest sugarcane burning and placing greater industrial restrictions, especially since the air pollution from the burning season has been associated with increased hospital admissions. ^4 , 14^

Although, in this study, the North Region was found to have the smallest PM _2.5_ concentration, other studies using different PM _2.5_ estimation methods described a greater air pollution burden in the region. ^15^ In fact, the North Region plays a crucial role in air pollution in Brazil. ^16^ The burning of biomass occurring in the Amazon, for instance, emits large amounts of PM _2.5_ , which not only impact areas close to the fires, but also cause illness and premature deaths in areas far away from them. ^16^ Furthermore, this region is also described as a socio-climatic hotspot, combining high levels of social and environmental vulnerability, enhancing the need for targeted socio-environmental and health interventions in the region. ^16^

Ultimately, although the numeric SBP increase value might seem dismissible, it must be viewed in the light of its impact on an extensive number of individuals and considered as a component of a continuum of exposures that have an additive effect on blood pressure. In addition, multiple biological pathways mediating the impact of PM _2.5_ on blood pressure have been proposed, including systemic inflammation, oxidative responses, and endothelial dysfunction. ^17^ Recent studies from China also implicate the activation of the hypothalamus-pituitary-adrenal axis in response to exposure to high levels of PM _2.5_ , culminating in significant increases in serum stress hormones (i.e., cortisol, cortisone, epinephrine, and norepinephrine). ^17 , 18^

This study’s limitations include those inherent to its ecological nature, its dependence on secondary data, and analysis on a state level, which can underestimate the effect in populations of industrialized metropolitan areas.
